# Comparison of shared decision making in patients undergoing hemodialysis and peritoneal dialysis for choosing a dialysis modality

**DOI:** 10.1186/s12882-021-02269-2

**Published:** 2021-02-23

**Authors:** Sepide Ghodsian, Mansour Ghafourifard, Akram Ghahramanian

**Affiliations:** 1 Department of Medical Surgical Nursing, Faculty of Nursing and Midwifery, Tabriz University of Medical Sciences, Tabriz, Iran; 2 Medical Education Research Center, Health Management and Safety Promotion Research Institute, Tabriz University of Medical Sciences, Tabriz, Iran

**Keywords:** Shared decision making, Hemodialysis, Peritoneal dialysis, Nursing care

## Abstract

**Background:**

Shared decision making (SDM) is recognized as the gold standard for patient-centered care. This study aimed to assess and compare the SDM among patients undergoing hemodialysis and peritoneal dialysis for choosing a dialysis modality.

**Methods:**

This is a cross-sectional study that was performed on 300 dialysis patients (218 HD and 82 PD) referred to two Dialysis Centers. Data were collected using demographic information and a 9-item Shared Decision Making Questionnaire (SDM-Q-9). The data were analyzed using ANOVA and independent t-test by SPSS software.

**Results:**

The mean SDM-Q-9 score in all samples (PD and HD) was 21.94 ± 15.08 (in a possible range of 0 to 45). Results of the independent t-test showed that the mean SDM-Q-9 score in PD patients (33.11 ± 10.08) was higher than HD patients (17.14 ± 74.24) (*p* < 0.001). The results showed a statistically significant difference in mean SDM-Q-9 score based on patients’ age, educational level, and income (*p* < 0.05).

**Conclusion:**

Implementing shared decision making and providing information on RRT should be started in the early stage of CKD. The health care providers should involve patients with CKD and their families in dialysis-related decisions and it should be started in the early stage of CKD.

## Introduction

Chronic kidney disease (CKD) is a disease with multiple causes that leads to an irreversible decrease in kidney functions and often leads to end stage kidney disease (ESKD) [1, 2]. Considering the increase in the elderly population and the increase in underlying diseases such as diabetes mellitus, hypertension, and obesity [3, 4], the global prevalence of CKD is also increasing 8% annually [5]; however, the growth of this disease in Iran is reported to be 12% [6]. It is estimated that the number of patients with ESKD will increase from 2.6 million in 2010 to 5.4 million in 2030 worldwide [7]. The prevalence of CKD in different countries varies by ethnic origin and social class and the risk of the disease in countries with lower socioeconomic status is 60% higher than in developed countries [5]. Control and treatment of this disease impose a high cost and a huge economic burden on the health care system, patients, and their families [8]. A recent study on the global burden of chronic kidney disease showed that treatment costs for CKD and costs related to the application of life-saving renal replacement techniques have raised during recent years [9].

Patients with ESKD need to choose one of the renal replacement therapies (RRT) choices including kidney transplantation or dialysis (hemodialysis or peritoneal dialysis), to survive and prevent uremia and other complications [10–12]. Among these treatments, hemodialysis is the most common treatment in Iran and the world [10, 13, 14]; about 87% of patients with ESKD undergo hemodialysis (HD) and only 13% of them are on peritoneal dialysis (PD) worldwide [13]. According to the latest statistics, there are 95% of HD patients and only 5% PD patients in Iran [15]. Choosing the type of RRT based on the patient’s conditions is considered a challenge for CKD patients [8]. Although kidney transplantation is the best choice for this disease, it cannot be performed for all patients due to the lack of easy access to donated kidneys, lack of information about transplanted kidneys, and taking into account the patient’s conditions [16]. Therefore, most patients with CKD should choose between home peritoneal dialysis (PD), in-center hemodialysis (IC-HD), and home hemodialysis (Home-HD. However, home HD is not usually offered and their choices are limited to in-center HD or home PD [17, 18].

Choosing the best treatment for patients requires cooperation between the patient and the healthcare team, which is called shared decision making (SDM) [19]. SDM is recognized as the gold standard for patient-centered care [20] and this approach has been proposed in more diseases, especially in chronic diseases [21]. SDM is a collaborative process in which patients, caregivers, family members, and even friends of the patient involve in health care decisions for patients, and the final decision is made based on the patient’s values, preferences, and conditions by taking into account the advantages and disadvantages of each treatment [19, 22]. The first step in this approach is to encourage the patient to engage in shared decision making, and this happens when sufficient information about each of the treatment options is made available to the patients and their family where the health care providers try to avoid imposing their opinions on patients choice [18].

Nurses have the most direct contact time with patients and they could influence patients’ knowledge and perception on shared decision making process [23]. Nephrology nurses could influence patients’ decisions by providing educations on modality selection. They can guide patients and their families for choosing the dialysis modality [24]. Providing education by nurses as a health care team could help patients to better understand chronic kidney diseases, weigh available renal replacement therapies, and decide on the modality selection [25]. During the shared decision process, nurses share the professional knowledge with the patients, and in turn, patients share their personal knowledge on their own daily life and health care. Finally, they discuss the choices and decide which dialysis modality (PD or HD) is best for him or her [26].

For providing a high quality care in patients with CKD, SDM approach has been suggested to choose the RRTs. For this, the healthcare teams need to explain all modalities to the patients and the advantages and disadvantages of each modality [27]. According to the literature review, many patients who have recently been diagnosed with ESKD do not have sufficient knowledge and information about RRT choices and are treated by one of RRT without even knowing its side effects [16]. Furthermore, previous studies show that many patients are not prepared enough to start dialysis [28] and they do not play an active role in choosing the type of dialysis [29, 30].

According to the literature review, SDM and patient involvement in treatment decisions are not still included in the national health system in Iran and it needs to be incorporated in clinical settings [31]. Home hemodialysis is not mainley offered in Iran and patients need to select the in-center hemodialysis or peritoneal dialysis. In terms of dialysis choice in Iran, the nephrologists generally decide the dialysis choice for patients and they usually suggest the in-center hemodialysis. However, some nephrologists may suggest the PD for patients or their family and there are no specific guidelines or decision aids for SDM in patients with CKD. Furthermore, there are no specific programs in hemodialysis units (including these two dialysis centers of our study) for predialysis education for the choice of dialysis. In some cases, nurses may suggest the PD modality for patients, but finally, the nephrologist decides the choice of dialysis modality. Moreover, many patients with emergency situations start renal replacement therapy with hemodialysis and continue on HD forever. There are notably more than 30,000 HD patients and only 1600 PD patients in Iran.

Although many patients can start and continue PD, most of patients with ESKD undergo hemodialysis [32, 33]. In this regard, a study by Erlang et al. [34] showed that PD was not introduced as one of RRTs for about 88% of dialysis patients. A literature review shows that PD leads to minimal disruption or change in the patient’s daily life and strengthens the patient’s self-care and independence [35]. PD is also considered as the best type of dialysis for patients who are on the waiting list for a kidney transplant [36]. In addition, PD is recommended in diabetic patients and patients with chronic heart failure, and this type of dialysis reduces the mortality rate of these patients [37, 38]. Other advantages of PD include the lower cost of this modality than other RRTs [39] and it is more cost-effective than HD [32]. Furthermore, previous studies revealed that PD leads to greater patient satisfaction [35, 40], as well as higher quality of life compared to HD [41]. However, PD has some limitations. This dialysis should be carried out every day and patients with PD has a greater risk of developing peritonitis, abdominal hernia, and disturbed body image. Furthermore, it is difficult to perform this type of dialysis in patients who have severe neurological problems and where there is no one to take care of patients with physical impairment or poor vision [42].

In order to involve patients in SDM regarding the type of dialysis treatment, patients and their caregivers should have a comprehensive understanding of the differences of dialysis methods and the impact of dialysis on their daily lives [43]. Patients who play an active role in SDM not only pay more attention to recommendations made by physicians and medical staff [44] but also have high self-confidence [45]. Moreover, SDM increases patient preparedness to overcoming the challenges of treatment-related problems and complications and reduces their worries [45]. Other advantages of the SDM approach include positive outcomes such as increasing the patient’s quality of life and maintaining patients’ independence [46]. However, when the patient is not involved in clinical decision-making and the choice of treatment is not selected based on the individual’s preferences and values, it may lead to conflict in decision-making [47] and may result in unpleasant consequences such as decreased patient’s motivation needed for treatment adherence, feeling of regret and guilt, and even blaming of the medical staff by the patient [27].

Although there have been studies on the effect of SDM in some diseases, a literature review shows that there are few studies on the field of dialysis [48]. Since SDM is an important factor in care of dialysis patients, we decided to assess and compare the SDM among patients undergoing hemodialysis and peritoneal dialysis for choosing the dialysis modality.

## Methods

### Design

This cross-sectional study was performed on 300 dialysis patients (218 HD and 82 PD) referred to two Dialysis Centers (Imam Reza and Sina Hospitals) affiliated to Tabriz University of Medical Sciences, Tabriz. All methods were performed in accordance with the relevant guidelines for cross-sectional studies (STROBE Statement).

### Sample and setting

There were 400 patients with HD and 90 patients with PD in these two dialysis centers. We used Krejcie & Morgan’s sampling table to determine the sample size of the study [29]. The patients were selected using a convenience sampling method. Inclusion criteria included patients aged over 18 years and who were undergoing hemodialysis or peritoneal dialysis. We included patients who were conscious and alert. We did not include the confused patients or those with cognitive problems based on the patient’s medical records.

### Data collection

Data were collected using demographic information and a 9-item Shared Decision Making Questionnaire (SDM-Q-9) from January to April 2020. SDM-Q was developed by Robinski et al. [43] in 2015 to probe the SDM process. The questionnaire consists of 9 questions that are answered based on a 6-point Likert scale (ranging from strongly disagree = 0, to strongly agree = 5). The possible score range is 0–45, with a higher score indicating a high SDM level. The overall score of SDM-Q-9 was standardized to 0–100.

The validity and reliability of this instrument have been investigated in previous studies with a Cronbach’s alpha of α = 94%, which indicates the high reliability of the scale [43]. In this study, the validity of the Persian version of the SDM-Q-9 was investigated by the content validity method. For this purpose, after being translated and re-translated by an English language expert, the questionnaire was given to ten faculty members of the faculty of Nursing and Midwifery and the questionnaire was revised based on their comments. Cronbach’s alpha of the Persian version of SDM-Q-9 was 98%.

The questionnaire was administered to the patient to complete it by self-administration method. However, a research coordinator was trained to interview patients who were unable to self-administer the SDM-Q-9 questionnaire, such as illiterate patients and patients with poor vision or physical impairment. She completed the questionnaire based on the participants’ responses. In addition, informations on patients’ clinical characteristics such as the cause of renal failure, duration of dialysis, and CKD comorbidities were also recorded.

### Data analysis

The collected data were analyzed using ANOVA and independent t-test by SPSS (ver. 21) software. The data were presented in tables with using descriptive analysis such as mean and standard deviation. The significance level was set at *p* < 0.05.

## Results

### Background characteristics of the patients

According to the result, the mean age of the subjects was 54.84 ± 14.98 years. A total of 168 patients were male and 132 were female. The majority of participants (84%) are married. The mean duration of hemodialysis treatment was 28.96 ± 24.86 months and the mean duration of peritoneal dialysis treatment was 38.68 ± 26.07 months (Table 1). Concerning CKD etiology, the most common cause was hypertension (24.6%), diabetes mellitus (18.7%), and a combination of diabetes mellitus and hypertension (17%).

**Table 1:** Demographic characteristics of patients (*N* = 300)

Variables	HD *N* = 218	PD *N* = 82	TOTAL *N* = 300
	Mean ± SD	Mean ± SD	Mean ± SD
Age (years)	56.44 ± 14.10	58.58 ± 15.46	54.84 ± 14.98
Duration of dialysis treatment (month)	28.96 ± 24.86	38.68 ± 26.07	31.62 ± 25.52
	N(%)	N(%)	N(%)
Gender			
Male	132 (60.6%)	36 (43.9%)	168 (56%)
Female	86 (39.4%)	46 (56.1%)	132 (44%)
Marital status			
Single	12 (5.5%)	16 (19.5%)	28 (9.3%)
Married	191 (87.6%)	61 (74.4%)	252 (84%)
Divorced	0 (0%)	1 (1.2%)	1 (0.4%)
Widow	15 (6.9%)	4 (4.9%)	19 (6.3%)
Education level			
Illiterate	55 (25.2%)	17 (20.7%)	72 (24%)
Elementary school	57 (26.2%)	16 (19.5%)	73 (24.3%)
Junior High school	55 (25.2%)	19 (23.2%)	74 (24.7%)
Diploma	36 (16.5%)	12 (14.6%)	48 (16%)
University	15 (6.9%)	18 (22%)	33 (11%)
Living in			
Urban	195 (89.4%)	66 (80.5%)	261 (87%)
Rural	23 (10.6%)	16 (19.5%)	39 (13%)
Income			
< 50 US dollars	114 (52.3)	20 (24.4)	134 (44.7)
50–100 US dollars	71 (32.6)	21 (25.6)	92 (30.7)
100–150 US dollars	25 (11.5)	35 (42.7)	60 (20)
> 150 US dollars	8 (3.7)	6 (7.3)	14 (4.6)

### Patients’ perception of shared decision making

Overall, the mean SDM-Q-9 score in all samples (PD and HD) was 21.94 ± 15.08 (in a possible range of 0 to 45). Results of the independent t-test showed that the mean SDM-Q-9 score in PD patients (33.11 ± 10.08) was higher than HD patients (17.14 ± 74.24), which was statistically significant (*p* < 0.001). Also, PD patients showed a higher score in all items of the SDM-Q-9 (*p* < 0.05).

Details of participants’ responses to each item of the SDM-Q-9 are shown in Table 2. According to the results of the questionnaire, the highest mean score (2.1 ± 46.77) among HD patients was related to item 1, “My physician told me that there are different methods of dialysis (HD and PD) to treat my disease” and the lowest mean score (1.80 ± 1.64) was related to Item 7, “My physician and I examined the types of dialysis (hemodialysis and peritoneal dialysis) “. In the case of PD patients, the highest mean score (3.42 ± 1.92) was related to Item 9“ My physician and I agreed to continue treatment” and the lowest mean score (3.37 ± 1.50) was related to Item 5 “My physician helped me understand all the information about HD and PD”.

**Table 2:** Item characteristics for the SDM-Q-9

Items (score for each item ranged 0 to 5)	HD mean ± sd	PD mean ± sd	total	COMPARISON OF HD & PD
1) My Doctor told me that there are different dialysis modalities (hemodialysis and peritoneal dialysis) for treating my medical condition	2.46 ± 1.77	3.87 ± 1.38	2.85 ± 1.78	T = − 6.51 *P* value = 0.0003
2) My Doctor made clear that a decision between hemodialysis and peritoneal dialysis needs to be made.	2.02 ± 1.66	3.78 ± 1.24	2.50 ± 1.74	T = − 8.65 *P* value = 0.0003
3) My Doctor wanted to know exactly how I Want to be involved in making the decision between hemodialysis and peritoneal dialysis	1.91 ± 1.63	3.53 ± 1.25	2.35 ± 1.69	T = − 8.14 *P* value = 0.0001
4) My Doctor precisely explained the advantages and disadvantages of hemodialysis and peritoneal dialysis	1.87 ± 1.58	3.59 ± 1.39	2.34 ± 1.71	T = − 8.66 *P* value = 0.0002
5) My Doctor helped me understand all of the information concerning hemodialysis and peritoneal dialysis	1.82 ± 1.60	3.50 ± 1.37	2.28 ± 1.71	T = − 8.36 *P* value = 0.0002
6) My Doctor asked me which dialysis treatment option (hemodialysis Or peritoneal dialysis) I prefer	1.89 ± 1.65	3.59 ± 1.30	2.36 ± 1.74	T = − 8.35 *P* value = 0.0002
7) My Doctor and I Thoroughly weighed the different dialysis treatment options (hemodialysis And peritoneal dialysis)	1.80 ± 1.64	3.58 ± 1.34	2.29 ± 1.75	T = − 8.75 *P* value = 0.0001
8) My Doctor and I Selected a dialysis treatment option (hemodialysis Or peritoneal dialysis) together	1.84 ± 1.66	3.68 ± 1.34	2.35 ± 1.77	T = − 8.95 *P* value = 0.0003
9) My Doctor and I Reached an agreement on how to proceed	2.09 ± 1.70	3.92 ± 1.42	2.59 ± 1.82	T = − 8.68 *P* value = 0.0002
SDM-Q-9 total score (ranged 0–45)	17.74 ± 14.24	33.08 ± 11.10	21.94 ± 15.08	T = −8.79 *P* value = 0.0001

### Comparison of SDM based on patients’ demographic

The results of the present study showed no statistically significant difference in mean SDM-Q-9 score based on gender, occupation, and living area (Urban or rural) (*P* > 0.05). However, education level had a significant effect on the mean SDM-Q-9 score (*P* < 0.05). In terms of total SDM-Q-9, Post hoc analysis showed that illiterate patients (17.14 ± 73.83) had a lower score than people with diploma degree (26.14 ± 96.75) and university education (26.96 14.75) (*P* < 0.05). Moreover, individuals with elementary education (18.14 ± 72.87) had a lower score than patients with diploma degree (26.96 ± 14.75) and university education (26.96 ± 14.75) (*P* < 0.05) (Table 3).

**Table 3:** Comparison of mean score of SDM-Q-9 based on patients characteristics (*N* = 300)

Variables	Mean	SD	*P*-value
Gender			
Male	21.91	14.72	*P* = 0.976
Female	21.96	15.58	T = 0.030
Marital status			
Married	21.39	14.80	*P* = 0.000003
Single	33.17	12.77	F = 9.97
Divorced	45	11.51	
Widow	11.42	11.43	
Education level			
Illiterate	17.73	14.83	*P* = 0.00002
Elementary school	18.72	14.87	F = 7.04
Junior High school	21.77	15.04	
Diploma	26.96	14.75	
University	26.96	14.75	
Living in			
Urban	22.47	15.22	*P* = 0.112
Rural	18.35	13.74	T = 0.067
Income			
< 50 US dollars	18.62	14.19	F = 6.13
50–100 US dollars	22.27	15.37	*P* = 0.00004
100–150 US dollars	26.75	15.31	
> 150 US dollars	30.85	12.13	

Concerning income level, the results show that monthly income had a significant effect on the mean SDM-Q-9 score (*P* < 0.05); Post hoc analysis showed that patients with a monthly income of lower than 50 Us dollars (18.14 ± 62.19) had a lower SDM score compared with patients with a monthly income of 100–150 US dollars (26.15 ± 75.31) and those with more than 150 Us dollars (30.85 ± 12.13) (*P* < 0.05). Moreover, there was a statistically significant difference between individuals with a monthly income of 10–20 million (22.27 ± 15.37) and those with an income level above 150 US dollars (30.85 ± 12.13) (*P* < 0.05).

Pearson correlation test showed a significant inverse relationship between age and SDM-Q-9 score, so that with increasing age, SDM score decreases (*p* < 0.05, r = − 0.382).

## Discussion

Providing person-centered care has recently been encouraged in the health care systems. Person-centered care is a holistic approach in which a good therapeutic relationship is established between patients and healthcare providers [49]. With regard to the nature of person-centered care, patient care decisions are made based on the needs, preference, and performance of the patients and they are involved in their treatment decisions. In fact, the patient plays an active role in the treatment process [50]. SDM is at the highest level of person-centered care and provide an opportunity for patients to engage in decisions and manage their health in an informed and effective way [48].

According to the results of the present study, two chronic diseases including hypertension and diabetes mellitus are the main cause of CKD in more than half of patients (59.3%). The literature review also reveals that these two diseases are the most important cause of CKD, which supports the results of the present study [51, 52].

The results showed no statistically significant difference in the mean SDM-Q-9 score based on sex, occupation, and place of residence. However, other variables such as patients’ education level, income, and age had a significant effect on the mean SDM-Q-9 score. According to the results, patients with higher education had a high SDM score. It seems that those with more education have more information about their disease and therefore try to participate consciously in care and clinical decisions. Therefore, they are more likely to take advantage of SDM. In line with our results, a study in Washington by Seo et al. [53] showed that people with higher health literacy levels were generally 2 times more likely to participate in treatment decisions than people with lower literacy levels. Mostafaie et al. also showed that people with higher education levels are more inclined to make shared decisions [54]. These results support the findings of our study.

The results of the present study also indicate that people’s income level also affects decision-making, so that people with higher income had more participation in clinical decisions. We could find no relevant study in this regard.

The results of the current study also showed an inverse relationship between the age and SDM-Q-9 scores so that with increasing age, the SDM score decreases. In this regard, Finderup et al. [49] found that although international guidelines recommend that patients should be involved in SDM, this is not always possible and older patients older than 65 years could not involved in the SDM process or unwilling to participate in care decisions {Finderup, 2019 #186}.

However, the mean age of the patients participating in this study was 54.84 ± 14.98 years and most of them could engage in the SDM on dialysis choice. Since there are no specific programs in hemodialysis units (including these two dialysis centers), most of the patients are not involved in SDM for the choice of dialysis.

Overall, the mean total SDM-Q-9 score among all participants (PD and HD) was 48.75 ± 35.52 out of the standardized score of 100. In a study carried out in Germany by Robinski et al. the mean total SDM-Q-9 score was 59.72 ± 24.33; which is higher than our results [43]. According to the results, the mean score of each item of SDM-Q-9 in our study is lower than two other studies carried out by Robinski et al. [43], and Finderup et al. [55]. The reason for these differences is related to the differences in the prevailing care approaches in different countries. Moreover, in developed countries such as Germany, there are dialysis staffs who work as dialysis coordinators. They play an important role in coordinating and involving the patient and their family in decisions related to choosing the dialysis. But, the person-centered care approach is not yet prevalent in our country, and most decisions are made by the medical team. Ambigapathy et al. believe that no patient decision should be made without patient involvement in health care systems [56].

Lee et al. reported that approximately 29% of CKD people did not complete the SDM process. They believed that this could be attributed to a lack of information on SDM, poor family support, and inappropriate initiation of SDM by the medical team. The ideal time to start SDM for RRT involves the early stage of CKD or initiation of stage V CKD, which can prevent the onset of emergency dialysis in hospitals, which is often carried out using a temporary hemodialysis catheter. The results of a recent study showed that the implementation of SDM increases the selection of PD and kidney transplantation rather than hemodialysis [57].

The results of the present study showed a statistically significant difference between PD and HD patients in terms of the mean SDM-Q-9 score; the mean SDM score in PD patients (73.52 ± 24.68 24) was higher than HD patients (39.43 ± 31.65). In a study in the United States, Zee et al. (2018) found that PD patients were more consciously involved in decision-making for the selection of the dialysis type compared to HD patients [58]. Also, in another study done by Robinski et al. [43], the results showed that PD patients had a higher SDM score (83.80 ± 24.42) than HD patients (61.36 ± 34.97), which is consistent with the findings of the present study. In Iran, there are more than 30,000 patients with HD while about 1600 patients with PD. It seems that most of the HD patients do not participate in the shared decision making process and they need more information to engage in decisions to choose one of the dialysis modality.

Finderup et al. [59] argued that factors impeding shared decision making in dialysis choice include providing biased or incomplete information on the dialysis options, not offering discussions and clarifications between patients and healthcare professionals, and lack of time before initiating dialysis. Thus, the choice between the choices of dialysis modalities should be selected based on patient preferences, and patients and their families should be encouraged to engage in the dialysis modality decision [58].

Based on the literature review, the choice of dialysis type varies depending on the preference and the lifestyle of each patient. In this regard, international guidelines recommend patients with CKD to involve in the SDM on dialysis modality selection based on the patient’s preferences and abilities [55, 60]. In some contexts, a physician or treatment staff have made decisions regarding HD and PD based on the patient’s clinical conditions, regardless of the patient’s preference and abilities [28, 61]. Similarly, in a study conducted in Germany in 2016, more than one-fourth of the HD patient stated that dialysis modality selection was made by a nephrologist without asking for their decisions and opinions [43]. A systematic review study showed that out of 18 studies, in 10 studies, patients and their families believed that they did not receive sufficient SDM-related information [62]. In a study in the United Kingdom, Durand et al. [63] found that more than half of patients with chronic kidney disease were unaware of all RRTs as a potential treatment and were reluctant to talk with their healthcare professionals about it. Balzer et al. also emphasize the importance of awareness of patients with ESKD and their relatives about dialysis methods to strengthen the patient’s empowerment and selection of home based dialysis. This study also reveals that PD patients, compared to HD patients, were more informed about the different methods before the initiation of dialysis and stated that they were provided with sufficient information [64].

This study has some limitations. This is the cross-sectional study which conducted in two educational hospitals, and it should be cautious when generalizing the results of this study to other dialysis centers. Moreover, we studied the SDM from the perspective of patients. Therefore, a larger multicenter study which includes the experience of healthcare professionals, patients, and their family on shared decision making in selecting a dialysis modality choice could provide further insight in this regard.

## Conclusion

Overall, the results of the present study showed a low and non-optimal SDM score in choosing the type of dialysis as compared to other studies. However, PD patients showed a better situation than HD patients and the SDM-Q-9 score was higher among PD patients compared with HD patients. Therefore, it is recommended to provide patients (especially HD patients) and their families with sufficient information on the type of RRT. Moreover, the health care team should involve patients and their families in dialysis-related decisions in a step-by-step manner. Implementing shared decision making and providing information on RRT should be started in the early stage of CKD. Moreover, it would be helpful to use a dialysis coordinator to improve the relationship between the patient and the medical team regarding the selection of dialysis modality. Another opportunity is to allow patients to have a plan of transitions and possible scenarios for transitions from one dialysis modality to another modality bases on the patients’ preference or clinical outcomes. Both patients and clinicians should follow this new paradigm of treatment that is to include the management of transitions between modalities toward better outcomes and survival in the career of CKD. The better approach should be to propose home dialysis whenever possible based on clinical and health economy sustainability.

The findings regarding shared decision making should be incorporated into the care of patients with CKD. The health care providers should involve patients with CKD and their families in dialysis-related decisions and this should be started in the early stage of CKD. Selecting a dialysis coordinator could improve the relationship between the patient and the medical team regarding the selection of dialysis modality.

## Data Availability

The datasets used and/or analyzed during the current study available from the corresponding author on reasonable request.
